# Nature's Nanotechnologists: Unveiling the Secrets of Diatoms

**DOI:** 10.1371/journal.pbio.0020306

**Published:** 2004-10-12

**Authors:** Jane Bradbury

## Abstract

Diatoms are unicellular algae with ornate silica shells. Their dazzling ability to build tiny structures could inspire applications in the semiconductor industry, drug delivery, and engineering

Diatoms, unicellular algae with ornate silica shells, have fascinated amateur and professional biologists ever since the invention of the microscope. But these days, diatoms and their exquisite shells are also attracting the attention of nanotechnologists who hope that diatoms will teach them how to make minute structures currently beyond the capabilities of materials scientists. And now these nanotechnologists, together with ecologists interested in the global carbon cycle—in which diatoms play a central role—have a genomic blueprint to help them in their studies: the annotated genome sequence of Thalassiosira pseudonana (http://genome.jgi-psf.org/diatom/).

## What Are Diatoms?

Diatoms, microalgae that are found in all aquatic and moist environments, first appeared more than 180 million years ago. Since then, diatom diversity has literally exploded; no one is sure how many living species there are—probably about 100,000—or why there are so many different types. Plant molecular biologist Chris Bowler (Ecole Normale Supérieure, Paris, France and Stazione Zoologica, Napoli, Italy) explains that molecular phylogeny and morphological studies suggest that diatoms originated ‘probably as the result of a eukaryote being invaded or engulfed by a photosynthetic eukaryote, most probably a red alga’.

The basic structure of all diatoms is similar: a single cell, often with a large vacuole, contained within a silica shell or frustule made of two overlapping halves or valves joined by girdle bands, which are also made of silica. The girdle bands form the rims of the two valves and allow unidirectional growth of the diatom during vegetative division. ‘The shell is rather like a Camembert cheese box or a petri dish’, explains marine ecologist Christian Hamm (Alfred Wegener Institute for Polar and Marine Research, Bremerhaven, Germany).

**Figure 1:**
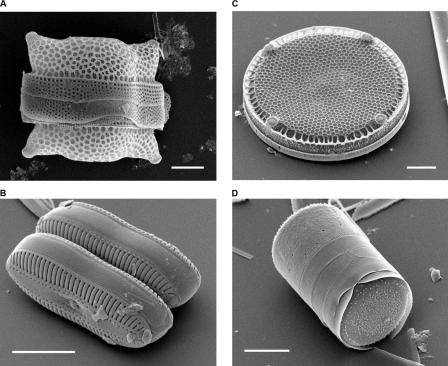
Scanning Electron Micrographs of Diatoms (A) Biddulphia reticulata. The whole shell or frustule of a centric diatom showing valves and girdle bands (size bar = 10 micrometres). (B) Diploneis sp. This picture shows two whole pennate diatom frustules in which raphes or slits, valves, and girdle bands can be seen (size bar = 10 micrometres). (C) Eupodiscus radiatus. View of a single valve of a centric diatom (size bar = 20 micrometres) (D) Melosira varians. The frustule of a centric diatom, showing both valves and some girdle bands (size bar = 10 micrometres). (Images courtesy of Mary Ann Tiffany, San Diego State University.)

There are only two main types of diatom: centric diatoms, which often have a circular symmetry, and pennate diatoms, which are usually bilaterally symmetrical. Nevertheless, diatom shells come in a dazzling array of forms and sizes (Figure 1; Box 1). ‘They can be circular, oval, stick-shaped, you name it, and range from several micrometres large to about a millimetre’, says ecologist Mary Ann Tiffany (San Diego State University, California, United States), who is using scanning electron microscopy to examine diatom valve formation as part of her graduate studies. ‘When a diatom divides, each daughter cell makes a new half shell’, explains Tiffany. The first stage of construction is the generation and deposition of silica nanospheres; the more ornate structures are built up from there. Both the finished shells, with their precise and reproducible nanometre-scale features, and the intermediate structures that lead up to the finished product, could be of interest to nanotechnologists, suggests Tiffany.

## Turning to Nature for Engineering Solutions

Richard Gordon, Professor of Radiology at the University of Manitoba in Winnipeg, Canada, somewhat accidentally laid the foundations of ‘diatom nanotechnology’ in 1988 when he was invited to give a lecture at an engineering conference. ‘I'm not an engineer’, explains Gordon, ‘but I knew engineers were interested in what was then called microfabrication so I told them about diatoms because they are so good at making small things’. Gordon, a keen diatom hobbyist, explained to his audience how diatoms could make a three-dimensional micro- or nanoscale structure for them without them lifting a finger. By contrast, says Gordon, ‘nanotechnology techniques then and now are tedious, involving painstakingly building three-dimensional structures up layer by layer’.

Such tedious techniques are currently used in the semiconductor industry. At present, explains Michael Sussman, Director of the Biotechnology Center at the University of Wisconsin-Madison (Madison, Wisconsin, United States), ‘features are etched onto circuit boards using light. However, the wavelength of light limits the smallest size that can be achieved, and for the next generation of faster computers, engineers need to get denser features onto computer chips than is possible with light etching’. Diatoms, says Sussman, ‘are natural-born lithographers in the nanometre range. If we could work out how diatoms lay down micro lines of silica, then we may be able to simulate it’. The proteins that diatoms use to direct silica deposition could be very useful to the semiconductor industry, says Sussman.

There are other ways in which diatoms could help us clumsy humans build nanoscale ‘widgets’. Molecular biologist Mark Hildebrand (Scripps Institution of Oceanography, San Diego, California, United States) is a member of a collaborative project trying to develop genetically engineered micro/nanodevices (also called GEMs). Already, engineers are using diatoms to help them build extremely sensitive sensors based on microfluidic devices, he explains. Hildebrand is also interested in the optical properties of diatoms. ‘Information processing technology is moving from electronically to optically based hardware, which allows more information to be carried and stored. Optical systems need materials with regularly repeating structures with features below the micrometre size range. These are very difficult to make by standard manufacturing techniques, but diatoms make structures like this all the time’.

It might also be possible to use diatom shells as delivery vehicles for drugs, suggests chemical engineer Tony Rogers, an assistant professor at Michigan Technological University (Houghton, Michigan, United States). ‘They have a uniform nanoscale pore structure and are chemically inert and biocompatible’. Rogers envisages loading diatoms with a drug that would then leach out into the blood stream at a rate dependent on the diatom species used. By incorporating ferromagnetic particles within the diatom structure, it might be possible to use a magnet to guide the drug to the right organ, he suggests.

Diatom structures are not just of interest to people interested in tiny objects. As Hamm comments, ‘in diatoms, Nature has solved many of the problems that engineers want to solve. For example, diatoms are particularly good at making lightweight but strong structures. Because it is possible to scale static structures like shells, diatoms can teach us how to make lightweight constructions for the aerospace and car industry’.

Some of the potential applications of diatoms can be investigated right now, using naturally occurring diatoms. In addition, subtle but important changes can be induced in diatoms by varying the amount of silica in their environment or changing the water flow. Gordon also envisages a device he calls a compustat, which would be used to select diatoms for a specific purpose. Diatoms taken from the sea, for example, would be individually examined using a computer-controlled microscope. ‘We would tell the computer what characteristics we were looking for, and it would go through the culture, zapping those diatoms furthest from the ideal with a laser beam. The culture would then be allowed to grow up again and the process repeated until we got the sort of diatoms we wanted’, says Gordon.

Gordon has not built a compustat yet—it may not work, he says, because we don't know how far we can push diatoms by forced evolution. And even if the compustat does work, to make the most of the nanotechnological potential of diatoms, we need to know exactly how diatoms make their shells. At present, all we know is that silicon transporters and a group of long-chain, polyamine-containing proteins called silaffins, which act as nucleation points for silica deposition, are involved. This is where the diatom sequencing project at the United States Department of the Environment's Joint Genome Initiative (JGI) at Walnut Creek, California, comes in.

## The First Diatom Is Sequenced

Daniel Rokhsar, Department Head for Computational Genomics at JGI, explains why his institute undertook the sequencing and computer annotation of the genome of T. pseudonana, a marine centric diatom ‘We believe that knowing this genome will help us to figure out how to mimic the processes that diatoms use to construct their very precise structures, and that we can then learn how to create similarly precise structures ourselves’. Also, he adds, diatoms are extremely important on an ecological level.

Oceanographer Ginger Armbrust (University of Washington, Seattle, Washington, United States), Principal Investigator on the sequencing project for T. pseudonana, explains further. Diatoms are responsible for between 25% and 40% of all the primary productivity of the oceans, she says. ‘They also keep the biological pump going. By fixing carbon dioxide and then sinking, diatoms draw carbon dioxide out of the atmosphere and take it into the deeper waters of the ocean, where it is retained for longer than it would be if the diatoms stayed near the surface’.

T. pseudonana, she continues, was chosen as the first diatom to sequence in part because it has a small genome, but mainly because it represents a cosmopolitan genus of diatoms and its physiology has been well studied. Once the primary sequence of the genome had been determined, molecular biologists, oceanographers, and ecologists from around the world gathered at JGI for a ‘genome jamboree’. ‘The first of these was in October 2002, a massive brainstorming session at which we all dug around in the genome for our favourite genes and tried to get a feel for what was there’, explains Bowler. ‘It was really refreshing to get the insights of oceanographers and ecologists into what this genome was telling us’.

Among other things, Armbrust and her collaborators are interested in finding out what the T. pseudonana genome can tell them about the difference between photosynthesis on land and in the sea. They also want to investigate how these organisms adapt to their environment. ‘Now that we have the genome’, says Armbrust, ‘we can investigate how gene expression varies at different places in the water column, for example. This will be the first time a eukaryotic genome has been interpreted in this ecological sort of way’.

## What About Silicon Metabolism and the Nanotechnology Dream?

‘One of the striking things about the T. pseudonana genome is that we can figure out quite a bit from it about how this diatom deals with organic materials, but it is hard to figure out what it is doing with silicon’, admits Rokhsar. ‘The only way we can really figure out what a gene is doing is by comparing it with known genes in other organisms, but because diatoms are so unique in their use of silicon, we don't have that option. We literally just have the parts list’.

To get a hook on which of the 10,000 or so T. pseudonana genes is important in silicon metabolism, Sussman is using microarrays to investigate how silicon concentrations affect gene expression patterns in the diatom. ‘There may be a few hundred genes whose expression changes in response to silicon stress’, he predicts, ‘and we can then focus on the role that these genes play in silicon metabolism’. In another approach, Hildebrand is purifying the proteins present in diatom shells. ‘Once we have isolated these proteins, we can get a little bit of protein sequence, and from there go back to the genome to pull the gene out’, he explains.

**Figure 3:**
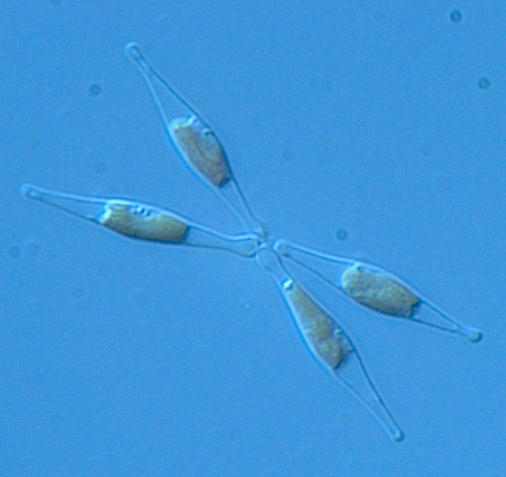
Light Micrograph of Phaeodactylum tricornutum This pennate diatom is the ‘lab rat’ of diatoms, and its genome sequence is currently being determined. (Image courtesy of Alessandra de Martino and Chris Bowler, Stazione Zoologica and Ecole Normale Supérieure.)

In an ideal world, the next step would be to see what effect genetically altering the expression of the proteins identified by Sussman and Hildebrand has on the silica shell of T. pseudonana. Unfortunately, this can't currently be done. ‘The only diatom we can genetically manipulate is Phaeodactylum tricornutum, a pennate diatom’, explains Bowler. P. tricornutum, he says, is the ‘lab rat’ of the diatom world but is much less important ecologically than T. pseudonana (Figure 3). Bowler has previously determined the size of P. tricornutum's genome and is now leading a JGI project that is 75% of the way through sequencing the P. tricornutum genome. ‘It will be critical to have this second genome’, notes Rokhsar, ‘because it will highlight what is unique to this group of organisms, and provide additional help in pulling out silicon metabolism genes’.

Once the details of silicon metabolism have been revealed, the stage should be set for nanotechnologists to harness diatom proteins for the manufacture of nanodevices. ‘Whether we use those proteins inside the diatom or in test tubes remains to be seen, but one way or another, diatoms are harbouring a secret that engineers need to learn about’, says Sussman. Hildebrand agrees, noting how ‘important it is that materials scientists recognise the incredible ability of biology to make structures that could perhaps be incorporated in the design of nanotechnological widgets’.

For Armbrust, it is the ecological insights are coming out of the T. pseudonana genome sequencing project—which is part of a bigger JGI program on algal genomics—that are most exciting. ‘Already, multiple little insights are encouraging us to think differently about how diatoms perceive their environment and survive in it. We have also seen many things we can't figure out at all right now. My heart lies in the ecology of these organisms, but if we can generate information that leads to spinoffs for nanotechnology, that will be fantastic’, she concludes.

**Figure 2:**
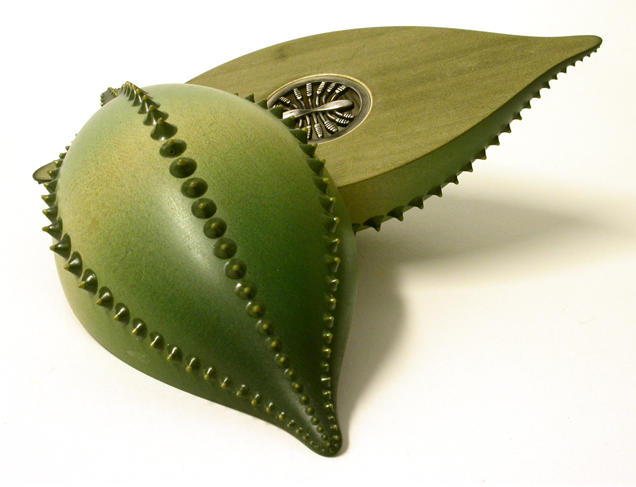
Diatoms in Art (Image courtesy of David Roberts, University of Wales, Bangor, UK.)

**Box 1. Diatoms in Art**

Diatoms don't inspire only biologists and engineers—artists, too, are fascinated by their intricate structures. Exquisite line drawings produced by zoologist Ernst Haeckel influenced the Art Nouveau movement, and more recently, wood-worker Louise Hibbert and jeweller Sarah Parker-Eaton have collaborated to produce three-dimensional objects based on diatoms and other plankton. ‘We both independently used marine biology as a source of inspiration for our art’, explains Parker-Eaton. ‘As a student, I often visited the Natural History Museum in London, where there were drawers of fascinating marine organisms that I could sketch’.

In January 2002, Hibbert and Parker-Eaton were invited to the marine laboratories at the University of Wales at Bangor (United Kingdom) by oceanographer David Thomas. ‘What we saw down the microscopes just blew our socks off’, says Parker-Eaton. ‘We could see how the plankton moved, the forms, the incredible different layers within the diatoms. What we particularly liked about diatoms was their complexity—they are totally unlike any other life form’.

Parker-Eaton and Hibbert translate what they see down the microscope into objects made of wood and silver, usually small enough to hold in the hand. Figure 2 shows a representation of Navicula sp. The main body is sycamore, the ‘blobs’ are resin, and the object is coloured with inks. The piece is held together by magnets but splits in half to reveal two silver inserts at its centre. More examples of these artists' work can be seen at http://www.louiseandsarah.com.

## Abbreviation

JGI: Joint Genome Initiative
